# Doctors in Council

**Published:** 1894-08-04

**Authors:** 


### The Hospital

AN INSTITUTIONAL JOURNAL OF

The Medical Sciences and Hospital Administration,

WITH

AN EXTRA NURSING SUPPLEMENT.

VOL. XVI.—No. 410.] [AUG. 4, 1894.

### DOCTORS IN COUNCIL.

THE Duke of Argyle has made the world familiar with the idea of the "reign of law," but civilised man is gradually submitting himself to a more concrete domination. On every side we see evidences of the beginning and wide extension of the "reign of the physiologist." The physiologist has come—he has come to stay—and, if the world is intelligent enough to value him at his real worth, he has come to reign.

It is not every worker who realises the true value and wide potentialities of his own work. A man need not be a very profound student of Shakespeare to perceive that the great dramatist, at any rate in the earlier days of his literary activity, hardly dreamt of the world-wide fame his work was destined to win, and the world-wide influence that work was ultimately to conquer. It is to some extent the same with the modern physiologist. If he be a man of logic he cannot but see that he is dealing with a science which is universal, and which, in its fullest normal development, must gradually illuminate and control the whole of every instructed man's life from the cradle to the grave. It too often happens, however, that the physiologist has little philosophy and less logic. In that case he sees in his science a mere auxiliary to therapeutics, instead of, as it truly is, the most glowing and world-embracing light of latter-day progress and achievement. What doctors cannot always see for themselves men of other learning can sometimes see for them. Speaking of that latest offspring of physiology, bacteriology, the Marquis of Salisbury uttered on a recent occasion these clear and far-seeing words: "I do not think that anyone who has watched the course of science will doubt that for a generation to come the investigation of these creatures (bacteria), which have been revealed to us by new methods of research and singularly patient labour, and upon which the lives of millions of human beings depend, will figure larger in the scientific field than any other object of study."

If doctors, by common consent of the learned and the practical alike, are to play so conspicuons and important a part in the all-round life of the coming generation, it is much to be desired that they should see clearly the nature and wide-reaching extent of their own work. It is equally imperative that the public, other than doctors, should clearly see and loyally encourage that progressive science which is destined more and more to deliver men from the dread of such "accidents" as "plague and pestilence," to give them reasonable assurance of competent health and a full life, and to animate them with the courage and high purpose which spring from such assurance. The Annual General Congress of the whole medical profession has been in session at Bristol during the past week. A glance at the programme of this British Medical Association and at some of the work undertaken by the association during the past twelve months, will show how physiology, in its widest sense, is the true physical gospel and salvation of the body of all mankind in every stage of our active life.

A mere catalogue of all the work done by the association during the year just past and summarised and revised at its annual session, would almost occupy the remaining space which can be allotted to this article. "Original research," as may be well imagined, has played a leading part. For all kinds of research dealing with the well-being of the human body in health and disease no fewer than thirty-six different researchers have been subsidised by the association and assisted in their work. The researchers have investigated questions of pure science, of common and rare diseases, of bodily degenerations, of new remedies and old remedies, of house sanitation, of atmospheric conditions modifying health, and the like. An association which keeps a body of thirty-six competent physiological investigators diligently at work, and measures with sound critical capacity their real or supposed results, deserves well of that civilised community in the vanguard of whose progress it marches.

A committee of the association has been investigating the physical and mental condition cf school children, and has recently reported upon fifty thousand individuals of this class. The work done by this committee is admirable. It shows what a large proportion of children even among the fairly prosperous classes vary in some points from what may be considered the normal standard for healthy, well developed childhood, and how many of them have therefore in themselves, and as no fault of their own, the elements of failure or even the elements of crime. Here, then, is an investigation which may lead to results of the very first consequence in many departments of our social and political life.

Another committee of the British Medical Association has had for its work during the year the guidance of the legislature in dealing with the very practical question of inebriates. It will be admitted that the question, What of a just and useful kind may we do with our chronic inebriates? is one of the most urgent difficulties of the hour. If medical science could put a stop to drunkenness, or, failing that, could say exactly what should be done with hopeless drunkards which should be universally felt to be "the right thing," it would render a service to civilisation which would be really great. Not yet can medicine say that it has solved the problem of habitual drunkenness. But this at any rate will be generally admitted, that if medicine cannot solve the problem it is likely for long to remain unsolved. The very fact that a large number of competent and honest men of science are diligently and patiently striving for the solution of the problem is at least hopeful if it be not greatly encouraging.

A bold attempt has been made by yet another committee to grapple with the ever-recurring question of the abuse of medical charities. The committee have at least something to show for the expenditure of their time and the money of their association. Th ey have propounded a scheme, for which we have not space even for an outline, which if carried out loyally by all the metropolitan hospitals, would at least reduce hospital abuse to a minimum. They have not been largely successful in persuading the hospitals to accept their scheme, but in that they have had any success at all, they may well take encouragement and resolve to persevere. The full programme of the association for the session week now in progress includes the formal discussion of such subjects as medicine, surgery, gynæcology, public medicine, psychology, pathology, ophthalmology, laryngology and otology, dermatology, and diseases of children. Each of these subjects, under the general presidency of Dr. Long Fox, has been discussed in its appropriate section by men of the first competence in Great Britain. It is thus that the lamp of medical science is annually trimmed and the fire of progress kept brightly burning.

There is some reason for general congratulation here. Medicine would stand still but for the enthusiasm and adventurousness of the few who lead and spur great assemblies of scientific men like the British Medical Association. What a calamity would that be for the world, and what a disgrace to the medical profession! The progress of the year is focussed at this annual congress; medical men realise where they stand or in what direction they are moving: and now at length the greater world for which all this work is done, and which is to be the ultimate rewarder of our scientific and medical gladiators, is beginning to look on with some glimmering of intelligent comprehension, with some faint warmth of appreciative enthusiasm, with some disposition to applaud and say "Well done!" It is "well done." Medicine at last is a great science and a noble art, and it can no longer be deprived of its well earned rights to recognition, leadership, and public honour.

